# Prevalence of prescription and effectiveness of analgesia for treating vaginal delivery pain

**DOI:** 10.1590/0034-7167-2023-0327

**Published:** 2024-11-22

**Authors:** Juan Ignacio Calcagno, Sarah Iribarren, Cristiane Flora Villarreal, Patricia Santos de Oliveira, Amado Nizarala de Ávila

**Affiliations:** I Maternidade Maria da Conceição de Jesus. Salvador, Bahia, Brazil; II University of Washington. Seattle, Washington, United States of America; III Universidade Federal da Bahia. Salvador, Bahia, Brazil; IV Fiocruz, Instituto Gonçalo Muniz. Salvador, Bahia, Brazil

**Keywords:** Labor Pain, Labor Obstetric, Pregnant Women, Obstetric Analgesia, Health Personnel, Dolor de Parto, Trabajo de Parto, Mujeres Embarazadas, Analgesia Obstétrica, Personal de Salud

## Abstract

**Objectives:**

to assess pain management during labor.

**Methods:**

a cross-sectional study was carried out by reviewing medical records and conducting postpartum interviews. Prevalence and effectiveness of analgesia were assessed.

**Results:**

the prevalence of non-pharmacological analgesia was 61.86% of 215 women in labor in Obstetric Center and 82.51% of 62 in midwife-led unit. Prevalence of severe pain, on the Visual Analogue Scale, before and after non-pharmacological analgesia, was from 92.16% to 64.04% (p=0.00) in Obstetric Center and from 85.96% to 52.63% (p=0.01) in midwife-led unit. Prevalence of pharmacological analgesia in Obstetric Centers was 15.81%, with no variation in severe pain (p=0.57). Patients’ request for analgesia was associated with education (p=0.00) and pain intensity (p=0.02).

**Conclusions:**

non-pharmacological analgesia improved pain intensity. Prevalence of pharmacological analgesic prescription was lower than that identified in developed countries. Pain management needs to consider the preferences and needs of women in labor.

## RESUMEN

**Objetivos:**

evaluar el manejo del dolor durante el parto.

**Métodos:**

estudio transversal realizado mediante revisión de historias clínicas y entrevistas posparto. Evaluamos la prevalencia y eficacia de la analgesia.

**Resultados:**

la prevalencia de analgesia no farmacológica fue 61,86% de 215 parturientas en el Centro Obstétrico y 82,51% de 62 en la sala dirigida por enfermeras. La prevalencia de dolor intenso, en la Escala Visual Analógica, antes y después de la analgesia no farmacológica, fue del 92,16% al 64,04% (p=0,00) y del 85,96% al 52,63% (p=0,01). La prevalencia de analgesia farmacológica en el Centro Obstétrico fue 15,81%, sin variación del dolor (p=0,57). La solicitud de analgesia se asoció con educación (p=0,00) e intensidad del dolor (p=0,02).

**Conclusiones:**

la analgesia no farmacológica mejoró el dolor. La prevalencia de analgesia farmacológica fue inferior que en países desarrollados. El manejo del dolor debe considerar preferencias y necesidades de las mujeres en trabajo de parto.

## INTRODUCTION

Maternal and perinatal outcomes can be improved through the implementation of quality practices, adequate use of preventive measures, technologies and interventions when necessary during childbirth and birth care^(1)^. Improving obstetric and neonatal indicators has been a global priority since the United Nations (UN) established the Millennium Development Goals in 2000^(2)^. Global efforts to improve these priority indicators have been significant, and in this context, Brazil has achieved institutional childbirth rates above 98%^(3,4)^. Despite these advances, Brazil’s indicators fall short of the UN targets, with a maternal mortality rate of 107 per 100,000 live births and an infant mortality rate of 13 per 1,000 live births in 2021. Additionally, Brazil presents one of the highest cesarean rates in the world at 55.7%^(5-9)^. These numbers indicate a need for improved quality of prenatal and perinatal care.

The Stork Network (RC - *Rede Cegonha*) was created by the Ministry of Health (MoH) in 2011 and updated in 2022 to improve service standards. RC aims to implement a model of childbirth care based on women’s needs, promoting vaginal delivery, offering technical support for professional qualification, and supporting the implementation of monitoring indicators across the country^(10,11)^. According to RC guidelines, labor and childbirth pain management should involve a combination of interventions, with availability of treatment using both pharmacological analgesia (PA) and non-pharmacological analgesia (NPA), tailored to each woman’s needs and preferences^(11)^. Despite the high rates of hospital childbirths in Brazil, the use of analgesia during labor and childbirth remains low, at just 4%^(12,13)^.

Pain, in addition to representing an important sign of labor, is defined as a subjective, unpleasant, sensorial and emotional experience that may or may not be associated with tissue damage^(14)^. Factors such as maternal age, parity, and ethnicity have been suggested as mediators of the experience of childbirth pain^(15,16)^. Furthermore, pain can also be influenced by anxiety, fear, stress, feelings of insecurity, and environmental factors, such as type of support provided, noise, quality of furniture, among others^(17-20)^.

The National Guidelines for Care to Vaginal Delivery, published by the Brazilian MoH in 2017, recommend that pregnant women should have access to pain relief techniques, including NPA and PA^(21)^. For labor pain management, healthcare professionals are advised to assess pregnant women’s knowledge on the subject, offer information about the different techniques available, and help them choose the most acceptable and appropriate options.

## OBJECTIVES

To assess obstetric care delivery during labor and childbirth, based on MoH guidelines with a specific focus on prevalence of prescription (PP) and effectiveness of NPA and PA techniques for managing labor and childbirth pain in women with low or moderate obstetric risk.

## METHODS

### Ethical aspects

The protocol for this study was approved by the *Escola Bahiana de Medicina e Saúde Pública* Research Ethics Committee. After explaining the objective of the study, all patients who agreed to participate signed the Informed Consent Form.

### Study design, period and place

This is a prospective, observational and cross-sectional study to assess the prevalence of techniques prescribed for pain management during labor and childbirth care for pregnant women at usual obstetric risk. We followed the guidance of Strengthening the Reporting of Observational studies in Epidemiology (STROBE, from EQUATOR) for publishing cross-sectional observational studies to report our results^(22)^.

This study was carried out at *Maternidade Maria da Conceição* (MMCJ), located in Salvador, Bahia, Brazil, between June 15 and October 31, 2022. MMCJ is a facility of the Brazilian Unified Health System (SUS - *Sistema Único de Saúde*) and was established in May 2021. Women in labor with usual obstetric risk, after risk screening by the nursing team, can choose where to receive care during labor and childbirth. There are two options available: Obstetric Center (OC), where women with any risk factors can receive care by midwives, obstetricians or a collaborative team of both; and the Midwife-Led unit (MLU), where only midwives provide care to healthy women with uncomplicated pregnancies, including those free from congenital malformations or fetal/placental diseases and who have received prenatal care. The MLU exclusively uses NPA techniques to relieve labor pain, whereas in OC, PA may be prescribed. If PA is needed, women admitted to MLU must be transferred to the OC. Obstetricians are legally responsible for both MLU and OC. However, in MLU, midwives manage women’s daily care and obstetricians are consulted in cases of complications or specific needs.

### Population and inclusion and exclusion criteria

During one year, 4,108 childbirths were performed, with 31.31% being cesarean sections. Therefore, 2,821 vaginal deliveries were recorded during the period. To calculate the sample size, Epi-Info 7.2 was used. Considering a 5% margin of error and a 95% Confidence Interval, it was calculated that 227 participants would be needed. The study included low-risk or intermediate-risk pregnant women hospitalized for vaginal delivery and cared for in OC or MLU. Excluded from the study were high-risk pregnant women, those with indications for a cesarean section, or postdactic pregnancy without labor.

### Study protocol

Data were collected through a questionnaire prepared by the main investigator based on literature reviews and administered at the bedside during the postpartum period. This questionnaire consisted of 40 questions, divided into six sections: sociodemographic information; obstetric history and comorbidities; characteristics of labor and childbirth; pain and pain management; presence of a companion; and complications. Race/color was measured using the classification from the Brazilian Institute of Geography and Statistics (IBGE - *Instituto Brasileiro de Geografia e Estatística*)^(23)^. Data extracted from medical records included time of admission, pain management, and prescription of interventions (e.g., when available, we recorded prescription of oxytocin, prostaglandin, amniotomy, episiotomy, and medical complications).

The convenience sampling technique was used to select participants from the daily census of hospitalized postpartum women, from July to October 2022, Monday to Friday. All who met the inclusion criteria were invited to participate.

### Analysis of results, and statistics

NPA techniques recorded included freedom to use different positions, ambulation, rhythmic breathing, relaxation techniques, hot shower or soak, massage or the use of a childbirth ball. These techniques aim to reduce pain and unnecessary administration of drugs that can interfere with the physiological processes that occur during labor and childbirth care. PA techniques recorded were regional epidural or epidural analgesia associated with subarachnoid.

The variables used in analysis were race/color, maternal age (years), marital status, education, obstetric history (pregnancies, childbirths, miscarriages, previous cesarean section), maternal comorbidities, gestational age at labor (weeks), duration of labor (hours) and birth (minutes), admission time, presence of a companion during labor and childbirth, place of childbirth, who performed the childbirth and pain intensity (measured using the Visual Analogue Pain Scale), asking patients to remember the intensity of pain before prescribing analgesia and after interventions (NPA and PA). Pain was categorized into 0-3 (mild), 4-7 (moderate) and 8-10 (severe). Fear was measured on a scale from zero (no fear) to ten (worst fear), asking patients to recall the intensity of fear during labor and childbirth. Additional data collected included patient request for analgesia, refusal of NPA and PA prescription by the multidisciplinary team, patient refusal to receive NPA or PA, time for administration after analgesia prescription, and type of NPA and PA prescription. Prescription of more than one type of NPA was categorized as multiple. The use of oxytocin, prostaglandin, episiotomy, amniotomy, perineal laceration and lithotomy position, obstetric and neonatal complications, and maternal and neonatal deaths were recorded.

A descriptive statistical analysis was performed (means, standard deviations, absolute values and proportions). For continuous variables, analysis of variance was used, and for categorical variables, the chi-square test was used. The analgesia PP rate (NPA and PA) was calculated using the total number of childbirths with analgesia prescription divided by the total number of childbirths, multiplied by a coefficient. Comparisons were carried out to assess differences between OC and MLU in pain management in women in labor and in the analgesic effectiveness of the prescribed methods. Odds Ratio, 95% Confidence Interval and p<0.05 were used as association variables. To assess statistical differences in the analgesic response before and after analgesic treatment (NPA and PA) of patients from the same unit, the McNemar test was used, considering one degree of freedom and p<0.05. Univariate logistic regression and multivariate logistic regression were used to analyze the determinants of analgesia prescription by medical staff and analgesia request by patients. Multivariate models were constructed using significant variables (p<0.1) in the univariate models. Statistical analysis was performed in Epi-Info 7.2.

## RESULTS

### Sociodemographic characteristics

A total of 277 patients participated, of which 215 (77.61%) were interviewed after childbirth care in OC and 62 (22.38%) in MLU. The mean age was 25 years (p 0.57). Women aged 18 to 34 years accounted for 85.20% of the sample. In OC, 54.88% of participants self-reported as mixed-race, compared to 45.16% in MLU. Black race/color was more common in MLU (53.23%) compared to OC (38.14%). White race/color was the least prevalent (Table 1). The most common level of education was high school (41.40% in the OC and 53.22% in MLU). Complete higher education was more common in MLU (17.74% versus 7.44% in OC). Regarding obstetric history, in both groups, a history of single pregnancy, single childbirth, absence of previous miscarriage and absence of previous cesarean section were more frequent. The occurrence of comorbidities was 40.00% in OC versus 11.29% in MLU. Statistically significant differences between the units were identified for black race/color (OR 1.84, 95%CI 1.04 - 3.26, p 0.03), education (OR 3.68, 95%CI 1.17 - 6, 13, p 0.01) and comorbidities (OR 0.31, 95%CI 0.12 - 0.77, p 0.00).

**Table 1:** Sociodemographic characteristics, Salvador, Bahia Brazil, 2022

Variables	MLU n= 62 (%)	OC n= 215 (%)	OR (95%CI) - *p* value
Race			
Mixed-race women	28 (45.16)	118 (54.88)	0.67 (0.38 - 1.19) - 0.17
Black women	33 (53.23)	82 (38.14)	1.84 (1.04 - 3.26) - 0.03
White women	1 (1.61)	15 (6.98)	0.21 (0.02 - 1.68) - 0.11
Education level			
Illiterate	0 (0.00)	2 (0.93)	--
Incomplete elementary school	8 (12.90)	41 (19.07)	0.62 (0.27 - 1.42) - 0.26
Elementary school	0 (0)	4 (1.86)	--
Incomplete high school	9 (14.29)	53 (24.65)	0.51 (0.23 - 1.12) - 0.09
High school	33 (53.22)	89 (41.40)	1.61 (0.91 - 2.84) - 0.09
Incomplete higher education	1 (1.61)	10 (4.65)	--
Higher education	11 (17.74)	16 (7.44)	3.68 (1.17 - 6.13) - 0.01
Age			
Mean age (min.-max.)	25.25	25.02	0.57
Marital status			
Married	45 (72.58)	146 (67.91)	1.25 (0.66 - 2.34) - 0.48
Single	17 (27.42)	69 (32.09)	0.79 (0.42 - 1.48) - 0.48
Obstetric history			
1st pregnancy	29 (46.77)	85 (39.53)	1.38 (0.78 - 2.44) - 0.26
2nd pregnancy	18 (29.03)	65 (30.23)	0.94 (0.50 - 1.75) - 0.85
3rd pregnancy	8 (12.90)	35 (15.81)	1.36 (0.67 - 2.74) - 0.39
Multiple pregnancy (>3)	2 (3.22)	30 (13.90)	--
History of a childbirth	35 (56.45)	104 (49.77)	1.37 (0.73 - 2.56) - 0.67
History of two childbirths	18 (29.03)	58 (26.98)	1.10 (0.59 - 2.05) - 0.74
History of three childbirths	8 (12.90)	34 (15.81)	0.78 (0.32 - 1.80) - 0.57
History > three childbirths	1 (1.61)	17 (7.91)	--
No previous miscarriages	50 (80.65)	168 (78.14)	1.16 (0.57 - 2.36) - 1.01
Only previous miscarriage	11 (17.74)	38 (17.67)	1.00 (0.47 - 2.10) - 0.99
More than one previous miscarriage	1 (1.61)	9 (4.19)	--
No prior cesarean section	62 (100)	193 (89.77)	--
Presence of comorbidity			
No comorbidity	55 (88.71)	155 (72.09)	0.31 (0.12 - 0.77) - 0.00
With comorbidity and types	7 (11.29)	62 (40.00)	--
Maternal syphilis	1 (14.29)	16 (25.81)	--
Gestational diabetes	0 (0.00)	11 (17.74)	--
Gestational hypertension	0 (0.00)	10 (16.13)	--
Anemia	1 (16.67)	9 (15.52)	--
Toxoplasmosis	0 (0.00)	5 (8.06)	--
Urinary tract infection	2 (28.57)	3 (4.84)	--
Asthma	2 (28.57)	2 (3.23)	--
Others	2 (28.57)	6 (9.66)	--

* *Higher education: university degree; MLU - midwife-led unit; OC - Obstetric Center; CI - Confidence Interval; OR - Odds Ratio.*

### Features of childbirth and birth care

For the current pregnancy, participants in this study arrived at the maternity ward with a mean of 39 weeks of gestation (p 0.63). About 95.3% of childbirths occurred between 37 and 41 weeks of gestational age (Table 2). The mean duration of labor and childbirth (from the onset of symptoms at home to birth registration at the unit) was 17.41 hours in MLU versus 14.97 hours in OC (p 0.18). The mean labor time under the supervision of the healthcare team was 9.75 hours in MLU and 8.63 in OC (p 0.32). For primiparous women, the mean time spent in the hospital unit was 10.40 hours for patients in OC and 11.74 for patients in MLU (p 0.40). In OC, 40.47% of patients were assisted during labor for more than eight hours, compared to 48.39% in MLU (OR 1.37, 95%CI 0.37 - 2.52, p .30). The mean childbirth time in minutes was 33.29 in MLU versus 31.55 in OC (p 0.51). Regarding who performed the childbirth, nurses were responsible for 80.87% of childbirths in MLU compared to 30.23% in OC (OR 12.00, 95%CI 5.74 - 26.00, p 0.00). In OC, childbirths were most frequently performed by a collaborative team of nurses and doctors (41.68%).

**Table 2:** Characteristics of labor and childbirth, Salvador, Bahia, Brazil, 2022

	MLU n= 62 (%)	OC n= 215 (%)	OR (95%CI) - *p* value
Mean gestational age	39.08 (37 - 41)	39.99 (33 - 42)	0.63
Mean total duration of labor and childbirth (h)	17.41 (4 - 73)	14.97 (2 - 61)	0.18
Mean labor and childbirth care time (h)	9.75 (1 - 48)	8.63 (1 - 42)	0.32
Labor care time < 3 (h)	13 (20.97)	70 (32.56)	0.54 (0.27 - 1.07) - 0.07
Labor care time 4 - 8 (h)	19 (30.65)	58 (26.98)	1.19 (0.64 - 2.20) - 0.57
Labor care time > 8 (h)	30 (48.39)	87 (40.47)	1.37 (0.77 - 2.52) - 0.30
Mean childbirth care time (min)	33.29 (2 - 60)	31.55 (1 - 180)	0.51
Who assisted childbirth			
Both (doctor and nurse)	7 (11.29)	90 (41.86)	0.17 (0.07 - 0.40) - 0.00
Nurse	52 (83.87)	65 (30.23)	12.00 (5.74 - 26.00) - 0.00
Doctor	2 (3.23)	45 (20.93)	--
First nurse and then doctor	1 (1.61)	15 (6.98)	--
Pain and fear scale and request for analgesia			
Mean pain on admission	9.09 (4 - 10)	8.93 (2 - 10)	0.52
Mild pain (0 - 3)	0 (0.00)	6 (2.79)	--
Moderate pain (4 - 7)	10 (16.13)	24 (11.16)	1.54 (0.68 - 3.40) - 0.29
Severe pain (8 - 10)	52 (83.87)	185 (86.05)	0.84 (0.38 - 1.83) - 0.66
Mean on fear scale (0 - 10)	5.34 (0 - 10)	6.70 (0 - 10)	0.11
Mild fear (0 - 3)^*^	7 (16.28)	19 (12.50)	0.97 (0.46 - 2.03) 0.93
Moderate fear (4 - 7)^*^	13 (3.23)	45 (29.61)	0.73 (0.28 - 1.88) 0.51
Severe fear (8 - 10)^*^	23 (53.49)	88 (57.89)	1.19 (0.60 - 2.36) 0.60
Request for analgesia^*^	16 (25.81)	62 (28.84)	0.85 (0.45 - 1.62) 0.64
Request for analgesia refused by the team^*^	6 (42.85)	13 (24.07)	--

* *Missing values were recorded in the variables fear, request for analgesia and team refusal; MLU - midwife-led units; OC - Obstetric Center; CI - Confidence Interval; OR - Odds Ratio.*

### Labor and childbirth and perception of pain and fear

The mean intensity of childbirth pain upon arrival at the unit was 9.09 in MLU and 8.93 in OC (p 0.52) on a scale of 0 to 10. Severe pain on admission was more frequently reported in OC (86.04 %) compared to MLU (83.87%). Despite experiencing intense pain, only 28.84% of OC patients and 25.81% of MLU patients requested analgesia (Table 3). In MLU, 42.85% of patients’ requests for analgesia were refused, compared to 24.07% in OC. The mean value of the fear scale upon arrival was 6.70 in OC versus 5.36 in MLU (p 0.11) on a scale from 0 to 10. No differences were identified in the levels of mild, moderate or severe fear between patients in these units. There were no associations found between fear and request for analgesia, nor between fear and absence of a companion during labor and/or childbirth.

**Table 3:** Pain management during labor and childbirth, Salvador, Bahia, Brazil, 2022

	MLU n= 62 (%)	OC n= 215 (%)	OR (95%CI) - *p* value
Multiple NPA methods prescribed	52 (82.51)	133 (61.86)	1.56 (0.55 - 4.38) - 0.39
Only one prescribed NPA method	5 (7.93)	20 (9.30)	0.63 (0.22 - 1.79) - 0.39
No NPA prescription	4 (6.45)	57 (26.51)	5.30 (1.84 - 15.29) - 0.00
NPA prescribed, but refused by patients	1 (1.61)	5 (2.32)	--
NPA prescription delay (h)	5.54 (0 - 22)	4.36 (0 - 22)	0.15
Pain improvement after NPA prescription	31 (54.39)	62 (40.52)	1.75 (0.94 - 3.23) - 0.07
Mean pain scale value after NPA (min.-max.)	7.54 (1 - 10)	8.12 (1 - 10)	0.10
Mild pain after NPA (0 - 3)	3 (5.26)	1 (0.66)	--
Moderate pain after NPA (4 - 7)	24 (42.11)	54 (35.29)	1.33 (0.71 - 2.48) - 0.36
Severe pain after NPA (8 - 10)	30 (52.63)	98 (64.05)	0.62 (0.33 - 1.15) - 0.13
Pain and PA scale			
No PA prescription	62 (100)	174 (80.93)	--
With PA prescription	--	34 (15.81)	--
PA prescribed, but refused by patient	--	7 (2.25)	--
Mean PA prescription delay (h)	--	11.65 (2 -23)	--
Pain improvement after PA	--	30 (88.23)	--
No improvement in pain after PA	--	4 (11.76)	--
Mean pain scale after PA	--	3.41 (0 - 10)	--
Mild pain after PA (0 - 3)	--	21 (38.24)	--
Moderate pain after PA (4 - 7)	--	9 (26.47)	--
Severe pain after PA (8 - 10)	--	4 (11.76)	--

*MLU - midwife-led units; OC - Obstetric Center; NPA - non-pharmacological analgesia; PA - pharmacological analgesia; CI - Confidence Interval; OR - Odds Ratio.*

### Pain management with pharmacological analgesia techniques in midwife-led units and Obstetric Centers

The PP of multiple NPA techniques was 82.51% in MLU versus 61.86% in OC (Table 3). In both units, the most prescribed techniques were showers with warm water, breathing techniques, massage, and childbirth balls (Table 4). Massage during labor was the only NPA statistically associated with a decrease in the frequency of severe pain (OR 0.50, 95%CI 0.27 - 0.91, p 0.02). Considering patients’ admission time to the maternity ward, the delay in prescribing NPA in hours was 5.54 in MLU, and 4.36 in OC (p=0.15). The mean pain after NPA prescription was 8.12 out of 10 in OC and 7.54 out of 10 in MLU (p=0.10). Severe pain upon admission to the maternity ward was compared with severe pain after NPA. Of a total of 62 patients in MLU, 58 (91.93%) were prescribed NPA, with one patient refusing after the prescription. Before the prescription, 49 (85.96%) of these 58 patients reported severe pain, and nine (15.78%) reported mild or moderate pain. After NPA treatment, 30 (52.63%) reported severe pain, while 27 (47.36%) reported a reduction to mild or moderate pain. There was a statistically significant decrease in severe pain before and after NPA prescription in MLU (χ2=5.80 > χc2=3.84, OR 0.55, p=0.01). In OC, of the 215 patients, 71.16% received a prescription and treatment with NPA. Of these, 141 (92.16%) reported initial intense pain, and 12 (7.84%) reported mild or moderate pain. After NPA prescription, 98 (64.04%) continued to report severe pain, while 55 (35.94%) reported a reduction to mild or moderate pain. One patient with initial mild to moderate pain reported severe pain after NPA. A statistically significant decrease in severe pain was identified before and after NPA prescription (χ2=36.86 > χc2=3.84, OR 0.39, p=0.00). As expected, no patient received PA in MLU. Additionally, no transfer from MLU to OC was recorded despite requests for PA.

**Table 4:** Prescription of evidence-based analgesic techniques and complications, Salvador, Bahia, Brazil, 2022

	MLU n= 62 (%)	OC n= 215 (%)	OR (95%CI) - *p* value
Non-pharmacological analgesia methods			
Hot shower bath	54 (94.74)	110 (71.90)	7.03 (2.08 - 23.71) - 0.00
Breathing techniques	42 (73.68)	105 (68.63)	1.28 (0.64 - 2.52) - 0.47
Massage	44 (77.19)	90 (58.82)	2.36 (1.17 - 4.75) - 0.01
Birthing stool	31 (54.39)	79 (51.63)	1.11 (0.60 - 2.05) - 0.72
Exercises	25 (45.86)	62 (40.52)	1.14 (0.62 - 2.12) - 0.66
Ball	25 (43.86)	36 (23.53)	2.53 (1.33 - 4.28) - 0.00
Music	9 (15.79)	24 (15.69)	1.00 (0.43 - 2.32) - 0.98
Aromatherapy	2 (3.51)	3 (1.96)	--
Methods of pharmacological analgesia			
Spinal + epidural	--	18 (42.94)	--
Epidural	--	14 (41.18)	--
Others	--	2 (5.88)	--
Scientific evidence-based interventions during labor and childbirth			
Prescription of oxytocin	16 (26.23)	67 (31.46)	0.77 (0.40 - 1.46) - 0.43
Episiotomy	0 (0.00)	1 (0.46)	--
Amniotomy	12 (19.35)	86 (40.19)	0.35 (0.17 - 0.70) - 0.00
Lithotomy position	0 (0.00)	(0.00)	--
Companion during labor and childbirth preferred by patients			
Companion during labor	61 (98.39)	196 (91.16)	5.91 (0.07 - 45.08) - 0.05
Companion during childbirth	61 (98.39)	191 (88.84)	7.63 (1.01 - 57.84) - 0.02
Laceration during childbirth			
Laceration	42 (67.74)	142 (66.36)	1.06 (0.58 - 1.94) - 0.83
Laceration - type 1	24 (57.14)	64 (46.04)	1.56 (0.77 - 3.13) - 0.20
Laceration - type 2	17 (40.48)	70 (52.36)	0.67 (0.33 - 1.35) - 0.26
Laceration - type 3	1 (2.38)	5 (3.60)	--
Laceration - type 4	0 (0.00)	0 (0.00)	--
Maternal complications			
Obstetric complication	4 (6.45)	13 (6.07)	1.06 (0.33 - 3.39) - 0.91
Postpartum hemorrhage	2 (50.00)	11 (76.47)	0.20 (0.01 - 2.38) - 0.19
Other obstetric complications	2 (50.00)	4 (23.53)	--
Neonatal complications			
Neonatal complication	3 (4.83)	27 (12.56)	0.35 (0.10 - 1.20) - 0.08
Respiratory discomfort	1 (33.33)	13 (48.00)	--
Risk of congenital syphilis	--	5 (17.86)	--
Jaundice	2 (66.67)	5 (17.86)	--
Other neonatal complications	0 (0.00)	5 (17.86)	--

*MLU - midwife-led units; OC - Obstetric Center; CI - Confidence Interval; OR - Odds Ratio.*

### Pain management with pharmacological analgesia techniques in Obstetric Centers

PA was prescribed to only 18.10% of patients, and 2.25% refused the medication due to concerns about its effects on labor progress. The mean delay in hours in PA prescription was 11.65 (Table 3). After NPA, only 34 (15.85%) patients were prescribed PA. Among these patients, 23 (67.64%) reported severe pain before PA, and 11 (32.36%) reported mild or moderate pain. After PA administration, six (26.08%) reported severe pain, and 17 (73.92%) reported a reduction to mild or moderate pain. None of the initial 11 patients with moderate and mild pain escalated to severe pain. The difference in severe pain before and after PA prescription was not statistically significant (χ2=0.31 < χc2=3.84, OR 1.21, p=0.57). The mean pain scale recorded after PA was 3.41 out of 10. No differences were found between race/color and prescription of NPA and PA for pain relief.

### Use of evidence-based interventions

The prescription of oxytocin ranged from 26.23% in MLU to 31.46% in OC (p 0.43). No childbirths were recorded in lithotomy position, and episiotomy was recorded only once (0.46%) in OC. Amniotomy was significantly more frequent in OC compared to MLU (OR 0.35, 95%CI 0.17 - 0.70, - p 0.00). Patients in the MLU had a companion more frequently during childbirth (98.39%) compared to OC patients (88.84%), and this difference was statistically significant (OR 7.63, 95%CI 1.01 - 57.84, p 0.02). The frequency of perineal lacerations during childbirth was high in both groups, with type 1 lacerations more frequent in MLU (57.14%) and type 2 laceration more frequent in OC (52.36%). No significant differences were found in the percentage of patients with a companion during labor, laceration, and complications.

### Variables associated with labor and childbirth pain management

Patients with incomplete elementary school (OR 0.30, 95%CI 0.12 - 0.74, p 0.00), shorter duration of labor, and childbirth care (less than three hours) (OR 0.50, 95%CI 0. 27 - 0.94, p 0.03) and moderate pain on arrival (OR 0.06, 95%CI 0.00 - 0.48, p 0.00) were associated with not requesting any type of analgesia for pain management. Variables associated with NPA prescription were a shorter duration of care until childbirth (less than three hours) (OR 0.26, 95%CI 0.14 - 0.46, p 0.00) and a longer duration of care (greater than eight hours) (OR 3.54, 95%CI 1.85 - 6.77, p 0.00). Variables associated with PA prescription included care provided by a collaborative team of doctors and nurses (OR 2.21, 95%CI 1.13 - 4.32, p 0.01), childbirth performed by the nursing team (OR 0.33, 95%CI 0.15 - 0.72, p 0.00), shorter duration of care until birth (less than three hours) (OR 0.04, 95%CI 0.00 - 0.34, p 0.00) and longer duration of care (greater than eight hours) (OR 7.46, 95%CI 3.29 - 16.89, p 0.00).

### Logistic regression

Each independent variable was assessed for association, identifying that the variables statistically associated with patients’ request for analgesia included incomplete elementary school (OR 0.33, 95%CI 0.13 - 0.84, p 0.02) and severe pain upon admission (OR 8.18, 95%CI 1.91 - 35.02, p 0, 00). Variables statistically associated with prescription of analgesics included childbirth performed by a collaborative team of doctors and nurses (OR 2.20, 95%CI 1.08 -4.49, p 0.02) and care duration exceeding eight hours (OR 7.45, 95%CI 3.27 - 16.99, p 0.00).

## DISCUSSION

The demographic characteristics of our study population, including race/color, age, education, and obstetric history, were similar to those reported in a study conducted in northeastern Brazil with 23,532 postpartum women^(24)^. This similarity suggests the external validity and representativeness of our collected data. Our findings were also compared with an analysis by the World Health Organization (WHO), which found that a low level of education was associated with underutilization of analgesia during vaginal delivery. However, the study did not establish whether the low use of analgesia was due to preferences of pregnant women or a lack of provision by healthcare professionals^(13)^. Another survey conducted with 99 postpartum women in Rio Grande do Norte found that approximately 80% of pregnant women who did not receive analgesia were unaware of its availability^(25)^. Although our study did not assess patients’ awareness of analgesia, we identified a significant association between lower levels of education and fewer requests for analgesia by patients.

In our study, only 21% of childbirths were exclusively attended by doctors. This contrasts with a survey in northeastern Brazil, where the proportion of vaginal deliveries attended by doctors ranged from 64.7% to 70.6%^(26)^. However, our study was conducted in a maternity hospital without an Intensive Care Unit for adults, which is not part of the medical residency teaching network in this specialty and focused exclusively on low and intermediate risk pregnant women, excluding those at higher risk of requiring a cesarean section. According to the Brazilian National Guideline for Care to Vaginal Delivery, childbirth for usual risk pregnancies can be attended by a doctor or nurse^(27)^. This may explain the higher proportion of childbirths attended by nurses or in collaboration with doctors found in our research. Our study also identified a high utilization of non-invasive techniques by the nursing team int the MLU, in line with WHO and MoH guidelines. This included a high prevalence of companions during labor and childbirth, low prescription rates of amniotomy and oxytocin, and high utilization rate of NPA methods. These non-invasive techniques are aimed at respecting the physiology of childbirth, and acknowledging women’s autonomy and the leading role of pregnant women in the birthing process^(28-30)^.

In this study, the mean delay in prescribing NPA was 4.36 hours in OC and 5.54 in MLU, while the delay for prescribing PA was 11.65 hours. In contrast, a study conducted in Israel assessed 575 vaginal deliveries and found that PA was prescribed to 94.4% of patients who requested pain management. Among the 33 patients without a PA prescription, 14 experienced delays due to the late arrival of an anesthetist, with mean waiting times ranging from five to 28 minutes^(31)^. This waiting time is considered a quality criterion for obstetric care. According to the Royal College of Anaesthetists in the United States, the waiting time for analgesic treatment should be 30 minutes, and in exceptional cases, up to 60 minutes^(32)^. In our research, we assessed the time between hospital admission and administration of analgesia. Anesthetists were located in the maternity ward, and their care depended on the request of obstetricians, which sometimes led to refusals of PA administration. Refusals are an indicator of the quality of obstetric care and the multidisciplinary team’s understanding of pain treatment. While NPA treatment guidelines do not specify the maximum acceptable time for starting therapy, it is generally understood that it should begin as early as possible and in accordance with maternal preference.

In the present study, patients who received multiple NPA interventions showed a reduction in intense pain, with massage being the only method independently associated with this effect. NPA techniques, known as mind-body interventions, are designed to induce calm, distract, and alleviate pain^(33)^. Our findings align with a Cochrane systematic review that identified massage as significantly reducing labor pain and improving emotional experience^(34)^. Similarly, a meta-analysis assessing the effects of NPA versus PA in childbirth pain management found that methods such as massage, water immersion, ambulation, and freedom to adopt different positions were associated with reduced PA prescriptions and higher levels of maternal satisfaction^(35)^. Contrasting the high prevalence of NPA in our results, Chassard *et al*. assessed 1,504 vaginal deliveries in France and found a NPA prevalence of just 19.5%, with a PA prevalence of 82.5%^(36)^. Another study in France assessed 9,231 childbirths and found a NPA PP of just 6.4%. Factors associated with NPA prescription included multiparity, institutions without a permanent capacity of anesthetists, and units with midwives. Most patients received PA (62.4%) or PA combined with NPA (32.2%). The combination of PA with NPA was associated with higher educational levels, public maternity wards, a preference for childbirth without PA expressed during prenatal care, and inadequate prenatal care^(37)^.

PA is an effective way of relieving pain during labor^(38-39)^. In developed countries, it is considered essential for pain management^(40)^. In France, PP of PA is among the highest in the world, with only one in 20 pregnant women not receiving PA for childbirth pain management^(37)^. In the United States, a study of 575,524 vaginal births also identified a high PP of PA (47.4%), but with notable disparities. PP of PA was less prevalent among racial minorities, uninsured, and low-income patients. Among pregnant women with health insurance, 75% received PA compared to 50% without health insurance^(41)^. In our research, only users of the Brazilian Unified Health System were assessed, primarily black and mixed-race women living in an economically disadvantaged region of the city of Salvador. In this context, pregnant women with a low level of education faced intense pain, possibly due to a lack of awareness about the benefits of PA. This may explain the low percentage of PA requests recorded during NPA treatment. In the present study, PA was frequently prescribed after eight hours of labor and requested by patients experiencing severe pain upon admission. Despite a significant decrease in pain intensity, no statistical differences were identified in severe pain levels before and after PA, likely due to the low number of prescriptions recorded. It is important to consider that early versus late initiation of labor analgesia does not result in clinically significant differences in maternal and neonatal outcomes^(42)^. Additionally, PA is not always associated with a positive childbirth experience, as it can reduce the sense of control for women. Furthermore, PA is more expensive than NPA, can delay the second stage of labor, and increase the likelihood of other interventions, such as instrumental delivery and cesarean section^(33,43)^.

### Study limitations

Some limitations of this study should be noted. First, patients’ perceptions about the adequacy of the infrastructure were not collected. While modern and comfortable maternity facilities are likely to positively impact patient experience, this has not been formally assessed. Second, the study did not explore patients’ prior knowledge, beliefs and preferences regarding different therapeutic modalities for pain relief, which may have influenced her decisions regarding pain management. Likewise, healthcare professionals’ beliefs, knowledge, concerns and needs regarding pain treatment were not assessed. Third, the study did not assess the prevalence of anxiety, depression or other mental health problems that may have affected the experience of childbirth pain. Fourth, recall bias may have influenced the accuracy of the data collected, and, to minimize this, interviews were conducted within 24 hours of childbirth. Fifth, it was not assessed whether the shifts were overcrowded with patients or whether the teams, made up of doctors and nurses, were adequately staffed for demand. Finally, PA-related adverse events and safety were not systematically recorded in the questionnaire.

### Contributions to health

According to the WHO, opioid analgesia is recommended for pain relief in women during labor and childbirth, along with non-pharmacological techniques. Intrapartum care must always respect women’s preferences, consider clinical evidence, effectiveness, complexity, diversity, equity, acceptability and viability of treatment options. Respecting women’s choices culminates in prioritizing individualized approaches that emphasize interpersonal relationships based on dialogue between women in labor and healthcare professionals.

We identified a high PP of multiple NPA methods and their effectiveness in reducing pain intensity, with massage alone being effective in reducing intense pain. Thus, in the high prevalence of NPA methods, the role of midwives in caring for women during labor and childbirth is significant.

Compared to analgesic practices in developed countries, PP of PA and the administration time of all types of analgesia studied here were below ideal standards. A low level of education was negatively associated with the request for analgesia. It is essential to understand that the painful experience is constructed from physiological, cultural, emotional, and social phenomena that make it unique and complex in its understanding, approach and treatment. Therefore, it is necessary to deepen knowledge of the factors associated with deficiencies related to analgesic treatment and initiate interventions aimed at educating patients, professionals and the public health system. Educational programs for patients, provided during prenatal care, would allow them to learn about analgesic possibilities, facilitating their active participation in choices and decision-making together with health teams. Clarification and active participation of patients could increase professionals’ adherence to prescriptions and reduce the time required to administer analgesia. Educational programs aimed at multidisciplinary teams are essential to train them to understand the complexity of pain management, considering its different components (physical, social, emotional and cultural), favoring an individualized approach that prioritizes pregnant women’s wishes and always emphasizing dialogue and collaboration between health teams. Multidisciplinary teams’ proactivity in the assessment and treatment of pain would provide improvements in clinical assessment, quality of care and, probably, the painful experience of women in labor. Hospital routines must be reassessed with the aim of developing institutional protocols based on pregnant women, defining criteria for prompt prescription and implementation of analgesia according to patients’ preferences. Finally, an administrative approach that strengthens the Brazilian Unified Health System structure and economic resources is also necessary, given that an increase in the availability of analgesia would cause an increase in costs and the need for effective hiring and training of adequately staffed and qualified multidisciplinary teams.

## CONCLUSIONS

This study highlights the complex nature of pain management during labor and childbirth and the underutilization of recommended pharmacological techniques for pain management. Although we observed a significant reduction in the proportion of patients with severe pain after NPA treatment and the effectiveness of massage as an isolated pain relief technique, there was a delay in the prescription and implementation of all types of analgesia. Furthermore, we identified a negative association between low education and patients’ request for analgesia. Additionally, the clinical staff did not always prescribe PA at patients’ requests, as recommended by the WHO, contributing to reduced PP of PA compared to developed countries.

It is crucial to train clinical staff and patients on the appropriate prescription of analgesia (time, type, and guidance for patients) and on its diversity and characteristics. Dialogue and collaborative work between multidisciplinary team and patients are fundamental. Furthermore, there is a lack of validated multidimensional instruments to measure the impact of pain during labor and childbirth. To deepen knowledge in the treatment of pain in pregnant women in labor, quantitative and qualitative studies are needed to assess barriers to pain assessment and treatment as well as develop multiple woman-centered interventions aimed at improving pain experience.
