# Supplementary material for: Prevalence of prescription and effectiveness of analgesia for treating vaginal delivery pain
Source: Rev Bras Enferm. 2024 Nov 22;77(5):e20230327. doi: 10.1590/0034-7167-2023-0327 (PMC11653877; doi:10.1590/0034-7167-2023-0327)
Supplement: Translation

# Translation: Prevalência de prescrição e efetividade da analgesia para o tratamento da dor do parto vaginal

**DOI:** 10.1590/0034-7167-2023-0327pt

**Authors:** Juan Ignacio Calcagno, Sarah Iribarren, Cristiane Flora Villarreal, Patricia Santos de Oliveira, Amado Nizarala de Ávila

**Affiliations:** I Maternidade Maria da Conceição de Jesus. Salvador, Bahia, Brasil; II Universidade de Washington, Seattle. Washington, Estados Unidos da América; III Universidade Federal da Bahia. Salvador, Bahia, Brasil; IV Fiocruz, Instituto Gonçalo Muniz. Salvador, Bahia, Brasil

**Keywords:** Dor do Parto, Trabalho de Parto, Gestantes, Analgesia Obstétrica, Pessoal de Saúde

## Abstract

**Objetivos:**

avaliar o manejo da dor no trabalho de parto.

**Métodos:**

estudo transversal realizado mediante revisão de prontuários e entrevistas pós-parto. Avaliaram-se prevalência e efetividade da analgesia.

**Resultados:**

a prevalência de analgesia não farmacológica foi 61,86% de 215 parturientes do Centro Obstétrico e 82,51% de 62 da ala liderada por enfermeiras. A prevalência de dor intensa, na Escala Visual Analógica, antes e depois da analgesia não farmacológica, foi de 92,16% para 64,04% (p=0,00) no Centro Obstétrico e de 85,96% para 52,63% (p=0,01) na ala das enfermeiras. A prevalência de analgesia farmacológica no Centro Obstétrico foi 15,81%, sem variação na dor intensa (p=0,57). O pedido de analgesia se associou à escolaridade (p=0,00) e à intensidade da dor (p=0,02).

**Conclusões:**

a analgesia não farmacológica melhorou a intensidade da dor. A prevalência de prescrição analgésica farmacológica foi inferior à de países desenvolvidos. O tratamento da dor precisa considerar preferências e necessidades das parturientes.

## INTRODUÇÃO

Os desfechos maternos e perinatais podem ser melhorados por meio de implementação de práticas de qualidade, utilização adequada de medidas preventivas, tecnologias e intervenções, quando necessárias, realizadas durante a assistência ao parto e ao nascimento^(1)^. A melhoria dos indicadores obstétricos e neonatais tem sido uma prioridade global desde que a Organização das Nações Unidas (ONU) estabeleceu os Objetivos de Desenvolvimento do Milênio em 2000^(2)^. Esforços globais para melhorar tais indicadores prioritários foram identificados e, neste contexto, o Brasil tem alcançado taxas de parto institucional superiores a 98%^(3,4)^. Apesar dos avanços, os indicadores do Brasil continuam apresentando resultados abaixo daqueles definidos pela ONU, incluindo taxa de mortalidade materna de 107 por 100.000 nascidos vivos e taxa de mortalidade infantil de 13 por 1.000 nascidos vivos em 2021. Além disso, o Brasil apresenta uma das maiores taxas cesariana do mundo, com 55,7%^(5-9)^. Esses números sugerem uma baixa qualidade da assistência pré-natal e perinatal.

A Rede Cegonha (RC) foi criada pelo Ministério da Saúde (MS), em 2011, e atualizada, em 2022, para melhorar os padrões de atendimento. A RC tem como objetivo implementar um modelo de assistência ao parto baseado nas necessidades das mulheres. Promove o parto vaginal, oferece suporte técnico para a qualificação profissional e apoia a implementação de indicadores de monitoramento em todo o país^(10,11)^. De acordo com as diretrizes da RC, o manejo da dor do trabalho de parto e parto deve envolver uma combinação de intervenções, com disponibilidade de tratamento com Analgesia Farmacológica (AF) e Analgesia Não Farmacológica (ANF), adaptadas às necessidades e preferências de cada mulher^(11)^. Apesar das altas taxas de partos hospitalares no Brasil, o uso de analgesia durante o trabalho de parto e parto permanece baixo, com apenas 4%^(12,13)^.

A dor, além de representar importante sinal do trabalho de parto, é definida como uma experiência subjetiva, desagradável, sensorial e emocional que pode, ou não, estar associada a dano tecidual^(14)^. Fatores como idade materna, paridade e etnia têm sido sugeridos como mediadores da experiência de dor de parto^(15,16)^. Ademais, a dor também pode ser influenciada por ansiedade, medo, estresse, sensação de insegurança, e fatores ambientais, como tipo de suporte fornecido, ruído, qualidade do mobiliário, entre outros^(17-20)^.

As Diretrizes Nacionais de Assistência ao Parto Normal, publicadas pelo MS do Brasil em 2017, recomendam que as gestantes devem ter acesso a técnicas de alívio da dor, incluindo ANF e AF^(21)^. Em relação ao manejo da dor do trabalho de parto, os profissionais de saúde são orientados a avaliar o conhecimento da gestante sobre o assunto, oferecer informações sobre as diferentes técnicas disponíveis e auxiliá-las a escolher as opções mais aceitáveis e adequadas.

## OBJETIVOS

Avaliar a assistência obstétrica, baseada nas orientações do MS, durante o trabalho de parto e parto, com foco específico na Prevalência de Prescrição (PP) e efetividade das técnicas de ANF e AF para o manejo da dor do trabalho de parto e parto em mulheres com risco obstétrico habitual ou intermediário.

## MÉTODOS

### Aspectos éticos

O protocolo deste estudo foi aprovado pelo Comitê de Ética em Pesquisa da Escola Bahiana de Medicina e Saúde Pública. Assim, após explicação do objetivo do estudo, todas as pacientes que aceitaram participar assinaram o Termo de Consentimento Livre e Esclarecido.

### Desenho, período e local do estudo

Estudo prospectivo, observacional e transversal, para avaliar a prevalência de técnicas prescritas para o manejo da dor durante o trabalho de parto e assistência ao parto de gestantes de risco obstétrico habitual. Seguimos a orientação da ferramenta *Strengthening the Reporting of Observacional studies in Epidemiology* (STROBE, do EQUATOR) para a publicação de estudos observacionais de tipo transversal para relatar os nossos resultados^(22)^.

Este estudo foi realizado na Maternidade Maria da Conceição (MMCJ), localizada na cidade de Salvador, Bahia, Brasil, entre 15 de junho e 31 de outubro de 2022. A MMCJ é uma entidade do Sistema Único de Saúde (SUS) criada em maio de 2021. Mulheres em trabalho de parto com risco obstétrico habitual, após triagem de risco pela equipe de enfermagem, podem escolher onde receber assistência durante o trabalho de parto e parto. Existem duas opções disponíveis: o Centro Obstétrico (CO), onde as mulheres com quaisquer fatores de risco podem receber cuidados por enfermeiras obstétricas, médicos obstétricos ou uma equipe colaborativa de ambos; e a ala liderada por enfermeiras obstétricas (ALEO), onde apenas enfermeiras obstétricas prestam cuidados a mulheres saudáveis com gestações não complicadas, incluindo livres de malformações congênitas ou doenças fetais/placentárias e que tenham realizado pré-natal. A ALEO utiliza apenas técnicas de ANF para alívio da dor do trabalho de parto, enquanto, no CO, pode ocorrer a prescrição da AF. Caso precise de AF, a mulher admitida na ALEO deve ser transferida para o CO. Os médicos obstetras são legalmente responsáveis tanto pela ALEO quanto pelo CO. No entanto, na ALEO, as enfermeiras obstétricas gerenciam o cuidado diário da mulher e os médicos obstetras são consultados em caso de complicações ou necessidades específicas.

### População e critérios de inclusão e exclusão

No período de um ano, foram realizados 4.108 partos, sendo que 31,31% foram por via cesariana. Sendo assim, foram contabilizados 2.821 partos vaginais no período. Para o cálculo do tamanho da amostra, utilizou-se o programa Epi-Info 7.2. Considerando uma margem de erro de 5% e um Intervalo de Confiança de 95%, calculou-se que 227 participantes seriam necessárias. Foram incluídas gestantes de baixo risco ou risco intermediário hospitalizadas em trabalho de parto vaginal e atendidas no CO ou ALEO. Foram excluídas gestantes de alto risco, com indicação de cesariana ou pós-datismo sem trabalho de parto deflagrado.

### Protocolo do estudo

Os dados foram coletados por meio de questionário elaborado pelo investigador principal com base em revisões da literatura e aplicado à beira do leito no pós-parto. Tal questionário foi composto por 40 questões, divididas em seis seções (sociodemográficas; histórico obstétrico e comorbidades; características do trabalho de parto e parto; dor e manejo da dor; presença de acompanhante; e complicações). A raça/cor foi mensurada utilizando a classificação do Instituto Brasileiro de Geografia e Estatística (IBGE)^(23)^. Os dados extraídos dos prontuários incluíram tempo de admissão, manejo da dor e prescrição de intervenções (por exemplo, quando disponível, registramos a prescrição de ocitocina, prostaglandina, amniotomia, episiotomia e complicações médicas).

Utilizou-se a técnica de amostragem por conveniência para selecionar as participantes do censo diário de puérperas hospitalizadas, no período de julho a outubro de 2022, de segunda a sexta-feira. Todas que atenderam aos critérios de inclusão foram convidadas a participar.

### Análise dos resultados e estatística

As técnicas de ANF registradas foram liberdade de usar diferentes posições, deambulação, respiração rítmica, técnicas de relaxamento, banho quente com chuveiro ou de imersão, massagem ou o uso de uma bola de parto. Essas técnicas visam reduzir a dor e a administração desnecessária de fármacos que podem interferir nos processos fisiológicos que ocorrem durante a assistência ao trabalho de parto e parto. As técnicas de AF registradas foram analgesia regional peridural ou peridural associada à subaracnóidea.

As variáveis utilizadas na análise foram raça/cor, idade materna (anos), estado civil, escolaridade, história obstétrica (gestações, partos, abortos, cesariana prévia), comorbidades maternas, idade gestacional no momento do trabalho de parto (semanas), duração do trabalho de parto (horas) e nascimento (minutos), tempo de admissão, presença de acompanhante durante o trabalho de parto e parto, local do parto, quem fez o parto e intensidade da dor (mensurada por meio da Escala Visual Analógica da Dor), solicitando-se a paciente que recordasse a intensidade da dor antes da prescrição da analgesia e após as intervenções (ANF e AF). A dor foi categorizada em 0-3 (leve), 4-7 (moderada) e 8-10 (intensa). O medo foi medido em uma escala de zero (sem medo) a dez (o pior medo), solicitando-se à paciente que recordasse a intensidade do medo durante o trabalho de parto e parto. Dados adicionais coletados incluíram solicitação de analgesia pela paciente, recusa da prescrição de ANF e AF pela equipe multiprofissional, recusa da paciente em receber ANF ou AF, tempo para a administração após a prescrição da analgesia e tipo de prescrição de ANF e AF. A prescrição de mais de um tipo de ANF foi categorizada em múltipla. Foram registrados o uso de ocitocina, prostaglandina, episiotomia, amniotomia, laceração perineal e posição litotômica, complicações obstétricas e neonatais, e óbitos maternos e neonatais.

Foi realizada uma análise estatística descritiva (médias, desvios padrão, valores absolutos e proporções). Para as variáveis contínuas, utilizou-se a análise de variância, e para as variáveis categóricas, usou-se o teste qui-quadrado. A taxa de PP de analgesia (ANF e AF) foi calculada utilizando-se o número total de partos com prescrição de analgesia dividido pelo número total de partos, multiplicado por um coeficiente. Comparações foram realizadas para avaliar diferenças entre o CO e a ALEO no manejo da dor das parturientes e na efetividade analgésica dos métodos prescritos. *Odds Ratio*, Intervalo de Confiança de 95% e p<0,05 foram utilizados como variáveis de associação. Para avaliar diferenças estatísticas na resposta analgésica antes e após o tratamento analgésico (ANF e AF) de pacientes da mesma unidade, foi utilizado o teste de McNemar, considerando um grau de liberdade e p<0,05. Regressão logística univariada e regressão logística multivariada foram utilizadas para analisar os determinantes da prescrição de analgesia pela equipe médica e a solicitação de analgesia pelos pacientes. Modelos multivariados foram construídos utilizando variáveis significativas (p<0,1) nos modelos univariados. A análise estatística foi realizada no programa Epi-Info 7.2.

## RESULTADOS

### Características sociodemográficas

Participaram 277 pacientes, das quais 215 (77,61%) foram entrevistadas após a assistência ao parto no CO e 62 (22,38%) na ALEO. A média de idade foi de 25 anos (p 0,57). Mulheres entre 18 e 34 anos representaram 85,20% da amostra. No CO, 54,88% das participantes se autorreferiram de raça/cor parda *versus* 45,16% na ALEO. A raça/cor preta foi a mais frequente na ALEO (53,23%), quando comparada com o CO (38,14%). A raça/cor branca foi a menos prevalente (Tabela 1). O nível de escolaridade mais frequente foi o ensino médio (41,40% e 53,22%, respectivamente). O ensino superior completo foi mais frequente na ALEO (17,74% *versus* 7,44%). Em relação aos antecedentes obstétricos, em ambos os grupos, história de gestação única, parto único, ausência de aborto prévio e ausência de cesariana prévia foram mais frequentes. A ocorrência de comorbidades foi de 40,00% no CO *versus* 11,29% na ALEO. Diferenças estatisticamente significativas entre as unidades foram identificadas para raça/cor preta (OR 1,84, IC95% 1,04 - 3,26, p 0,03), escolaridade (OR 3,68, IC95% 1,17 - 6,13, p 0,01) e comorbidades (OR 0,31, IC95% 0,12 - 0,77, p 0,00).

**Tabela 1:** Características sociodemográficas, Salvador, Bahia, Brasil, 2022

Variáveis	ALEO n= 62 (%)	CO n= 215 (%)	OR (IC95%) - Valor de *p*
Raça			
Mulheres pardas	28 (45,16)	118 (54,88)	0,67 (0,38 - 1,19) - 0,17
Mulheres pretas	33 (53,23)	82 (38,14)	1,84 (1,04 - 3,26) - 0,03
Mulheres brancas	1 (1,61)	15 (6,98)	0,21 (0,02 - 1,68) - 0,11
Nível de escolaridade			
Analfabeta	0 (0,00)	2 (0,93)	--
Ensino fundamental incompleto	8 (12,90)	41 (19,07)	0,62 (0,27 - 1,42) - 0,26
Ensino fundamental	0 (0)	4 (1,86)	--
Ensino médio incompleto	9 (14,29)	53 (24,65)	0,51 (0,23 - 1,12) - 0,09
Ensino médio	33 (53,22)	89 (41,40)	1,61 (0,91 - 2,84) - 0,09
Nível superior incompleto	1 (1,61)	10 (4,65)	--
Nível superior	11 (17,74)	16 (7,44)	3,68 (1,17 - 6,13) - 0,01
Idade			
Idade média (mín.-máx.)	25,25	25,02	0,57
Estado civil			
Casada	45 (72,58)	146 (67,91)	1,25 (0,66 - 2,34) - 0,48
Solteira	17 (27,42)	69 (32,09)	0,79 (0,42 - 1,48) - 0,48
História obstétrica			
1ª gravidez	29 (46,77)	85 (39,53)	1,38 (0,78 - 2,44) - 0,26
2ª gravidez	18 (29,03)	65 (30,23)	0,94 (0,50 - 1,75) - 0,85
3ª gravidez	8 (12,90)	35 (15,81)	1,36 (0,67 - 2,74) - 0,39
Gravidez múltipla (>3)	2 (3,22)	30 (13,90)	--
Histórico de um parto	35 (56,45)	104 (49,77)	1,37 (0,73 - 2,56) - 0,67
Histórico de dois partos	18 (29,03)	58 (26,98)	1,10 (0,59 - 2,05) - 0,74
Histórico de três partos	8 (12,90)	34 (15,81)	0,78 (0,32 - 1,80) - 0,57
Histórico > a três partos	1 (1,61)	17 (7,91)	--
Nenhum aborto anterior	50 (80,65)	168 (78,14)	1,16 (0,57 - 2,36) - 1,01
Único aborto anterior	11 (17,74)	38 (17,67)	1,00 (0,47 - 2,10) - 0,99
Mais de um aborto anterior	1 (1,61)	9 (4,19)	--
Sem cesariana prévia	62 (100)	193 (89,77)	--
Presença de comorbidade			
Sem comorbidade	55 (88,71)	155 (72,09)	0,31 (0,12 - 0,77) - 0,00
Com comorbidade e tipos	7 (11,29)	62 (40,00)	--
Sífilis materna	1 (14,29)	16 (25,81)	--
Diabetes gestacional	0 (0,00)	11 (17,74)	--
Hipertensão gestacional	0 (0,00)	10 (16,13)	--
Anemia	1 (16,67)	9 (15,52)	--
Toxoplasmose	0 (0,00)	5 (8,06)	--
Infecção do trato urinário	2 (28,57)	3 (4,84)	--
Asma	2 (28,57)	2 (3,23)	--
Outras	2 (28,57)	6 (9,66)	--

* *Ensino superior: diploma universitário; ALEO - Ala Liderada por Enfermeiras Obstétricas; CO - Centro Obstétrico; IC - Intervalo de Confiança; OR - Odds Ratio.*

### Características da assistência ao trabalho de parto e parto

Ao considerar a gestação atual, as participantes deste estudo chegaram à maternidade com média de 39 semanas de gestação (p 0,63). Aproximadamente 95,3% dos partos foram registrados entre 37 e 41 semanas de idade gestacional (Tabela 2). A duração média do trabalho de parto e parto (desde o início dos sintomas no domicílio até o registro de nascimento na unidade) foi de 17,41 horas na ALEO *versus* 14,97 horas no CO (p 0,18). O tempo médio de trabalho de parto até o parto, supervisionado pela equipe de saúde, foi de 9,75 horas na ALEO e de 8,63 no CO (p 0,32). Considerando apenas as primíparas, o tempo médio decorrido, em horas, sob cuidados na unidade hospitalar, foi de 10,40 para as pacientes no CO e de 11,74 para as pacientes na ALEO (p 0,40). No CO, o porcentual de pacientes que foram assistidas durante o trabalho de parto até o parto, por mais de oito horas, foi de 40,47% *versus* 48,39% na ALEO (OR 1,37, IC95% 0,37 - 2,52, p 0,30). O tempo médio de parto em minutos foi de 33,29 na ALEO *versus* 31,55 no CO (p 0,51). Ao avaliar quem realizou o parto, as enfermeiras foram responsáveis por 80,87% dos partos na ALEO *versus* 30,23% no CO (OR 12,00, IC95% 5,74 - 26,00, p 0,00). No CO, os partos foram realizados com maior frequência por equipe colaborativa de enfermeiros e médicos (41,68%).

**Tabela 2:** Características do trabalho de parto e parto, Salvador, Bahia, Brasil, 2022

	ALEO n= 62 (%)	CO n= 215 (%)	OR (IC95%) - Valor de *p*
Média de idade gestacional	39,08 (37 - 41)	39,99 (33 - 42)	0,63
Média total de duração de TP e P (h)	17,41 (4 - 73)	14,97 (2 - 61)	0,18
Tempo médio de assistência de TP e P (h)	9,75 (1 - 48)	8,63 (1 - 42)	0,32
Tempo de assistência de TP < 3 (h)	13 (20,97)	70 (32,56)	0,54 (0,27 - 1,07) - 0,07
Tempo de assistência de TP 4 - 8 (h)	19 (30,65)	58 (26,98)	1,19 (0,64 - 2,20) - 0,57
Tempo de assistência de TP > 8 (h)	30 (48,39)	87 (40,47)	1,37 (0,77 - 2,52) - 0,30
Tempo médio de assistência ao P (min)	33,29 (2 - 60)	31,55 (1 - 180)	0,51
Quem assistiu o P			
Ambos (médico e enfermeiro)	7 (11,29)	90 (41,86)	0,17 (0,07 - 0,40) - 0,00
Enfermeiro	52 (83,87)	65 (30,23)	12,00 (5,74 - 26,00) - 0,00
Médico	2 (3,23)	45 (20,93)	--
Primeiro enfermeiro e, depois, médico	1 (1,61)	15 (6,98)	--
Escala de dor e medo e solicitação de analgesia			
Média de dor na admissão	9,09 (4 - 10)	8,93 (2 - 10)	0,52
Dor leve (0 - 3)	0 (0,00)	6 (2,79)	--
Dor moderada (4 - 7)	10 (16,13)	24 (11,16)	1,54 (0,68 - 3,40) - 0,29
Dor severa (8 - 10)	52 (83,87)	185 (86,05)	0,84 (0,38 - 1,83) - 0,66
Média na escala de medo (0 - 10)	5,34 (0 - 10)	6,70 (0 - 10)	0,11
Medo leve (0 - 3)^*^	7 (16,28)	19 (12,50)	0,97 (0,46 - 2,03) 0,93
Medo moderado (4 - 7)^*^	13 (3,23)	45 (29,61)	0,73 (0,28 - 1,88) 0,51
Medo severo (8 - 10)^*^	23 (53,49)	88 (57,89)	1,19 (0,60 - 2,36) 0,60
Solicitação de analgesia^*^	16 (25,81)	62 (28,84)	0,85 (0,45 - 1,62) 0,64
Solicitação de analgesia recusada pela equipe^*^	6 (42,85)	13 (24,07)	--

* *Valores faltantes foram registrados nas variáveis medo, solicitação de analgesia e recusa da equipe; ALEO - Ala Liderada por Enfermeiras Obstétricas; CO - Centro Obstétrico; IC - Intervalo de Confiança; OR-Odds Ratio; TP-Trabalho de Parto; P-Parto.*

### Trabalho de parto e parto e percepção de dor e medo

A intensidade média de dor de parto na chegada na unidade foi 9,09 na ALEO e 8,93 no CO (p 0,52), em uma escala 0 a 10. Dor intensa na admissão foi mais frequentemente relatada no CO (86,04%), quando comparada com a ALEO (83,87%). Mesmo com dor intensa, apenas 28,84% das pacientes do CO e 25,81% das pacientes da ALEO solicitaram analgesia (Tabela 3). Na ALEO, 42,85% dos pedidos de analgesia das pacientes foram recusados *versus* 24,07% no CO. O valor médio da escala de medo na chegada foi de 6,70 no CO *versus* 5,36 na ALEO (p 0,11), em uma escala 0 a 10. Não foram identificadas diferenças no medo leve, moderado ou grave entre as pacientes nessas unidades. Não foram verificadas associações entre medo e solicitação de analgesia, e entre medo e ausência de acompanhante durante o trabalho de parto e/ou parto.

**Tabela 3:** Manejo da dor durante o Trabalho de Parto e Parto, Salvador, Bahia, Brasil, 2022

	ALEO n= 62 (%)	CO n= 215 (%)	OR (IC95%) - Valor de *p*
Múltiplos métodos de ANF prescritos	52 (82,51)	133 (61,86)	1,56 (0,55 - 4,38) - 0,39
Apenas um método de ANF prescrito	5 (7,93)	20 (9,30)	0,63 (0,22 - 1,79) - 0,39
Sem prescrição de ANF	4 (6,45)	57 (26,51)	5,30 (1,84 - 15,29) - 0,00
ANF prescrita, mas recusada pela paciente	1 (1,61)	5 (2,32)	--
Atraso de prescrição de ANF (h)	5,54 (0 - 22)	4,36 (0 - 22)	0,15
Melhoria de dor após prescrição de ANF	31 (54,39)	62 (40,52)	1,75 (0,94 - 3,23) - 0,07
Valor médio da escala de dor após ANF (mín.-máx.)	7,54 (1 - 10)	8,12 (1 - 10)	0,10
Dor leve após ANF (0 - 3)	3 (5,26)	1 (0,66)	--
Dor moderada após ANF (4 - 7)	24 (42,11)	54 (35,29)	1,33 (0,71 - 2,48) - 0,36
Dor severa após ANF (8 - 10)	30 (52,63)	98 (64,05)	0,62 (0,33 - 1,15) - 0,13
Escala de dor e AF			
Sem prescrição de AF	62 (100)	174 (80,93)	--
Com prescrição de AF	--	34 (15,81)	--
AF prescrita, mas recusada pela paciente	--	7 (2,25)	--
Média de atraso de prescrição de AF (h)	--	11,65 (2 -23)	--
Melhora de dor após AF	--	30 (88,23)	--
Sem melhoria de dor após AF	--	4 (11,76)	--
Média na escala de dor após AF	--	3,41 (0 - 10)	--
Dor leve após AF (0 - 3)	--	21 (38,24)	--
Dor moderada após AF (4 - 7)	--	9 (26,47)	--
Dor severa após AF (8 - 10)	--	4 (11,76)	--

*ALEO - Ala liderada por Enfermeiras Obstétricas; CO - Centro Obstétrico; ANF - Analgesia Não Farmacológica; AF - Analgesia Farmacológica; IC - Intervalo de Confiança; OR - Odds Ratio.*

### Manejo da dor com técnicas de analgesia farmacológica na ala liderada por enfermeiras obstétricas e no Centro Obstétrico

A PP de múltiplas técnicas de ANF foi de 82,51% na ALEO *versus* 61,86% no CO (Tabela 3). Em ambas as unidades, as técnicas mais prescritas foram banho de chuveiro com água morna, técnicas de respiração, massagem e bola de parto (Tabela 4). A massagem durante o trabalho de parto foi a única ANF estatisticamente associada à diminuição da frequência de registro de dor intensa (OR 0,50, IC95% 0,27 - 0,91, p 0,02). Considerando o tempo de admissão da paciente na maternidade, o atraso na prescrição da ANF em horas na ALEO foi de 5,54, e no CO, de 4,36 (p 0,15). A média de dor após a prescrição da ANF foi de 8,12 em 10 no CO e de 7,54 em 10 na ALEO (p 0,10). A dor intensa na admissão na maternidade foi comparada com a dor intensa após ANF. De um total de 62 pacientes na ALEO, 58 (91,93%) receberam prescrição de ANF e, após a prescrição, uma paciente recusou. Dessas 58 pacientes, 49 (85,96%) referiram dor intensa, e nove (15,78%), dor leve ou moderada antes da prescrição. Após o tratamento com ANF, 30 (52,63%) referiram dor intensa, e 27 (47,36%) relataram redução para dor leve ou moderada. A dor intensa, antes e após a prescrição da analgesia na ALEO, apresentou uma diminuição estatisticamente significativa (χ2=5,80 > χc2=3,84, OR 0,55, p=0,01). No CO, de um total de 215 pacientes, 71,16% receberam prescrição e tratamento com ANF. Dessas, 141 (92,16%) referiram dor intensa inicial, e 12 (7,84%), dor leve ou moderada. Após a prescrição da ANF, 98 (64,04%) continuaram relatando dor intensa, e 55 (35,94%) relataram redução para dor leve ou moderada. Uma paciente com dor inicial leve a moderada relatou dor intensa após ANF. Foi identificada uma diminuição estatisticamente significativa na dor intensa antes e após a prescrição da ANF (χ2=36,86 > χc2=3,84, OR 0,39, p=0,00). Como esperado, nenhuma paciente recebeu AF na ALEO. No entanto, nenhuma transferência da ALEO para o CO foi registrada, apesar da solicitação de AF.

**Tabela 4:** Prescrição de técnicas analgésicas baseadas em evidências e complicações, Salvador, Bahia, Brasil, 2022

	ALEO n= 62 (%)	CO n= 215 (%)	OR (IC95%) - Valor de *p*
Métodos de Analgesia Não Farmacológica			
Banho quente de chuveiro	54 (94,74)	110 (71,90)	7,03 (2,08 - 23,71) - 0,00
Técnicas de respiração	42 (73,68)	105 (68,63)	1,28 (0,64 - 2,52) - 0,47
Massagem	44 (77,19)	90 (58,82)	2,36 (1,17 - 4,75) - 0,01
Cavalinho	31 (54,39)	79 (51,63)	1,11 (0,60 - 2,05) - 0,72
Exercícios	25 (45,86)	62 (40,52)	1,14 (0,62 - 2,12) - 0,66
Bola	25 (43,86)	36 (23,53)	2,53 (1,33 - 4,28) - 0,00
Música	9 (15,79)	24 (15,69)	1,00 (0,43 - 2,32) - 0,98
Aromaterapia	2 (3,51)	3 (1,96)	--
Métodos de Analgesia Farmacológica			
Raquidiana + peridural	--	18 (42,94)	--
Peridural	--	14 (41,18)	--
Outros	--	2 (5,88)	--
Intervenções baseadas em evidências científicas durante o TP e P			
Indicação de ocitocina	16 (26,23)	67 (31,46)	0,77 (0,40 - 1,46) - 0,43
Episiotomia	0 (0,00)	1 (0,46)	--
Amniotomia	12 (19,35)	86 (40,19)	0,35 (0,17 - 0,70) - 0,00
Posição litotômica	0 (0,00)	(0,00)	--
Acompanhante no TP e P de preferência da paciente			
Acompanhante durante TP	61 (98,39)	196 (91,16)	5,91 (0,07 - 45,08) - 0,05
Acompanhante durante P	61 (98,39)	191 (88,84)	7,63 (1,01 - 57,84) - 0,02
Laceração durante o P			
Laceração	42 (67,74)	142 (66,36)	1,06 (0,58 - 1,94) - 0,83
Laceração - tipo 1	24 (57,14)	64 (46,04)	1,56 (0,77 - 3,13) - 0,20
Laceração - tipo 2	17 (40,48)	70 (52,36)	0,67 (0,33 - 1,35) - 0,26
Laceração - tipo 3	1 (2,38)	5 (3,60)	--
Laceração - tipo 4	0 (0,00)	0 (0,00)	--
Complicações maternas			
Complicação obstétrica	4 (6,45)	13 (6,07)	1,06 (0,33 - 3,39) - 0,91
Hemorragia pós-parto	2 (50,00)	11 (76,47)	0,20 (0,01 - 2,38) - 0,19
Outras complicações obstétricas	2 (50,00)	4 (23,53)	--
Complicações neonatal			
Complicação neonatal	3 (4,83)	27 (12,56)	0,35 (0,10 - 1,20) - 0,08
Desconforto respiratório	1 (33,33)	13 (48,00)	--
Risco de sífilis congênita	--	5 (17,86)	--
Icterícia	2 (66,67)	5 (17,86)	--
Outras complicações neonatais	0 (0,00)	5 (17,86)	--

*ALEO - Ala Liderada por Enfermeiras Obstétricas; CO - Centro Obstétrico; IC-Intervalo de Confiança; OR - Odds Ratio; TP-Trabalho de Parto; P- Parto.*

### Manejo da dor com técnicas de analgesia farmacológica no Centro Obstétrico

A AF foi prescrita para apenas 18,10% das pacientes, e 2,25% recusaram a medicação por preocupação com seus efeitos na evolução do trabalho de parto. O atraso médio, em horas, na prescrição da AF, foi de 11,65 (Tabela 3). Após ANF, apenas 34 (15,85%) pacientes receberam prescrição de AF. Dessas pacientes, 23 (67,64%) relataram dor intensa antes da AF, e 11 (32,36%) referiram dor leve ou moderada. Após AF, seis (26,08%) declararam dor intensa, e 17 (73,92%) relataram redução para dor leve ou moderada. Nenhuma das 11 pacientes iniciais com dores moderada e leve alterou sua escala de dor para dor intensa. A diferença entre dor intensa, antes e após a prescrição da AF, não foi estatisticamente significativa (χ2=0,31 < χc2=3,84, OR 1,21, p=0,57). A média da escala de dor registrada após AF foi de 3,41 em 10. Não foram encontradas diferenças entre raça/cor e prescrição de ANF e AF para o alívio da dor.

### Uso de intervenções baseadas em evidências

A prescrição de ocitocina variou de 26,23% na ALEO a 31,46% no CO (p 0,43). Não foi registrado parto em posição litotômica, e a episiotomia foi registrada apenas uma vez (0,46%) no CO. A amniotomia foi significativamente mais frequente no CO, quando comparada à ALEO (OR 0,35, IC95% 0,17 - 0,70, - p 0,00). As pacientes da ALEO tiveram mais frequentemente acompanhante durante o parto (98,39%) *versus* paciente do CO (88,84%), e a diferença foi estatisticamente significativa (OR 7,63, IC95% 1,01 - 57,84, p 0,02). A frequência de laceração durante o parto foi alta em ambos os grupos. A laceração tipo I foi mais frequente na ALEO (57,14%), e a laceração tipo 2 foi mais frequente no CO (52,36%). Não foram encontradas diferenças significativas no porcentual de pacientes com acompanhante durante o trabalho de parto, laceração e complicações.

### Variáveis associadas ao manejo da dor do trabalho de parto e parto

Pacientes com ensino fundamental incompleto (OR 0,30, IC95% 0,12 - 0,74, p 0,00), tempo de trabalho de parto e assistência ao parto inferior a três horas (OR 0,50, IC95% 0,27 - 0,94, p 0,03) e dor moderada na chegada (OR 0,06, IC95% 0,00 - 0,48, p 0,00) se associaram à não solicitação de nenhum tipo de analgesia para o manejo da dor. As variáveis associadas à prescrição de ANF foram tempo de assistência até o parto inferior a três horas (OR 0,26, IC95% 0,14 - 0,46, p 0,00) e tempo de assistência superior a oito horas (OR 3,54, IC95% 1,85 - 6,77, p 0,00). As variáveis associadas à prescrição de AF foram assistência realizada por equipe colaborativa de médicos e enfermeiros (OR 2,21, IC95% 1,13 - 4,32, p 0,01), parto realizado pela equipe de enfermagem (OR 0,33, IC95% 0,15 - 0,72, p 0,00), tempo de assistência inferior a três horas (OR 0,04, IC95% 0,00 - 0,34, p 0,00) e tempo de assistência superior a oito horas (OR 7,46, IC95% 3,29 - 16,89, p 0,00).

### Regressão logística

Foi avaliada a associação de cada variável independente, identificando que as variáveis estatisticamente associadas à solicitação de analgesia pela paciente incluíram ensino fundamental incompleto (OR 0,33, IC95% 0,13 - 0,84, p 0,02) e dor intensa na admissão (OR 8,18, IC95% 1,91 - 35,02, p 0,00). As variáveis estatisticamente associadas à prescrição de analgésicos foram o parto realizado por equipe colaborativa de médicos e enfermeiros (OR 2,20, IC95% 1,08 -4,49, p 0,02) e o tempo de assistência superior a oito horas (OR 7,45, IC95% 3,27 - 16,99, p 0,00).

## DISCUSSÃO

Os dados demográficos da população estudada, incluindo raça/cor, idade, escolaridade e antecedentes obstétricos, foram semelhantes aos relatados em estudo realizado na região Nordeste brasileira em 23.532 puérperas^(24)^. Essa semelhança poderia sugerir a validade externa e a representatividade dos dados coletados. Nossos dados também foram contrastados com uma análise realizada pela Organização Mundial da Saúde (OMS), que identificou que o baixo grau de escolaridade apresentava associação com a falta de prescrição de analgesia durante o parto vaginal. Porém, a pesquisa não estabeleceu se o baixo uso de analgesia refletia desejo das gestantes ou uma necessidade não atendida pelos profissionais^(13)^. Outra pesquisa realizada com 99 puérperas no Rio Grande do Norte registrou que aproximadamente 80% das gestantes sem prescrição de analgesia desconheciam a sua disponibilidade^(25)^. Na nossa pesquisa, não foi avaliado o conhecimento das pacientes sobre a analgesia, mas identificamos uma associação significativa entre o menor grau de instrução e a menor solicitação de analgesia pela paciente.

Em nosso estudo, apenas 21% dos partos foram assistidos exclusivamente por médicos. Esses achados contrastam com uma pesquisa que registrou que, na região Nordeste do Brasil, a proporção de partos vaginais assistidos por médicos variou de 64,7% a 70,6%^(26)^. Porém, nosso estudo foi realizado em uma maternidade sem Unidade de Terapia Intensiva para adultos, a qual não faz parte da rede de ensino para médicos residentes da especialidade e que teve como foco de interesse apenas gestantes de riscos baixo e intermediário, evitando a análise de uma população de pacientes com maior risco de evolução para a cesariana. Segundo a Diretriz Nacional de Assistência ao Parto Normal, o parto de risco habitual pode ser realizado por médico ou enfermeiro^(27)^. Isso poderia explicar a maior proporção de parto realizado pela enfermagem ou em conjunto com os médicos, apurada na nossa pesquisa. No presente estudo, também foi identificada elevada utilização de técnicas não invasivas pela equipe de enfermagem da ALEO, baseadas nas orientações da OMS e do MS, tais como elevada prevalência de acompanhante durante o trabalho de parto e parto, baixa PP de amniotomia e ocitocina, e elevada PP de métodos de ANF. Essas técnicas não invasivas são medidas focadas no respeito à fisiologia do parto, no resgate da autonomia da mulher e no protagonismo da gestante no processo parturitivo^(28-30)^.

Neste estudo, o atraso médio em horas na prescrição de ANF foi de 4,36 no CO e de 5,54 na ALEO, e de 11,65 na prescrição de AF. Diferentemente desses resultados, pesquisa realizada em Israel avaliou 575 partos vaginais. A PP de AF foi de 94,4% das pacientes que solicitaram o manejo da dor; entre as 33 pacientes sem prescrição de AF, em 14, ocorreu por demora na chegada do anestesista; o tempo médio de espera para a chegada do anestesista variou de cinco a 28 minutos^(31)^. Esse tempo de espera é considerado um critério de qualidade da assistência obstétrica. Segundo o Colégio Real de Anestesiologia dos Estados Unidos, o tempo de espera para o tratamento analgésico deveria ser de 30 minutos e, em casos excepcionais, até 60 minutos^(32)^. Na nossa pesquisa, foi avaliado o tempo entre admissão hospitalar e administração da analgesia. Os anestesistas estavam alocados na maternidade, e a sua assistência dependia da solicitação dos obstetras. Sendo assim, foram registradas recusas quanto à administração de AF. A recusa é um indicador da qualidade da assistência obstétrica e do nível de compreensão da equipe multiprofissional sobre o tratamento da dor. Conquanto, as guias de tratamento com ANF não estabelecem o tempo máximo aceitável para o início da terapêutica, entende-se que seria o mais precocemente possível e de acordo com a preferência materna.

No presente estudo, as pacientes que receberam múltiplas intervenções de ANF apresentaram redução da dor intensa, sendo a massagem o único método que, isoladamente, esteve associado a este efeito. As técnicas de ANF conhecidas como intervenções mente-corpo são projetadas para induzir calma, distrair e aliviar a dor^(33)^. Nossos achados foram consistentes com revisão sistemática da Cochrane, que identificou que a massagem reduz significativamente a dor do trabalho de parto e melhora a experiência emocional^(34)^. Também alinhada com nossos resultados, uma meta-análise avaliou os efeitos da ANF versus AF no manejo da dor de parto. Métodos como massagem, imersão em água, deambulação e liberdade para adotar diferentes posições estavam associados à redução da prescrição de AF, com níveis mais elevados de satisfação materna^(35)^. Contrastando a elevada PP de ANF identificada em nossos resultados, Chassard *et al*. avaliaram 1.504 partos vaginais na França, com PP de ANF de apenas 19,5% e PP de AF de 82,5%^(36)^. Também na França, outro estudo avaliou 9.231 partos com PP de ANF de apenas 6,4%. Os fatores associados à prescrição de ANF foram multiparidade, instituições sem lotação permanente de anestesistas e unidades com parteira. A maioria das pacientes recebeu AF (62,4%) ou AF associada à ANF (32,2%). A combinação de AF com ANF foi associada ao elevado nível educacional, à maternidade pública, ao relato de preferência durante o pré-natal sobre o parto sem AF e ao pré-natal com assistência inadequada^(37)^.

A AF é uma forma eficaz de alívio da dor durante o trabalho de parto^(38-39)^. Nos países desenvolvidos, ela é considerada essencial para o manejo da dor^(40)^. Na França, a PP de AF é uma das maiores do mundo, sendo que apenas uma de cada 20 gestantes não recebe AF para o manejo da dor do parto^(37)^. Nos Estados Unidos, um estudo avaliou 575.524 partos vaginais e também identificou uma elevada PP de AF (47,4%), porém houve iniquidades. A PP de AF foi menor em minorias raciais, pacientes sem plano de saúde e com baixa renda. Das gestantes com plano de saúde, 75% receberam AF *versus* 50% sem plano de saúde^(41)^. Na nossa pesquisa, foram avaliadas apenas usuárias do SUS, principalmente de cores preta e parda, moradoras de uma região economicamente desfavorecida da cidade de Salvador. Neste contexto, gestantes com baixo nível de escolaridade enfrentaram dor intensa por possivelmente desconhecerem os benefícios da AF. Isto poderia justificar o baixo porcentual de solicitações de AF registrado durante o tratamento com ANF. No presente estudo, a prescrição de AF foi frequentemente realizada após oito horas de trabalho de parto e solicitada por pacientes com dor intensa na admissão. Apesar de registrada expressiva diminuição da intensidade da dor, não foram identificadas diferenças estatísticas na dor intensa antes e após a AF, provavelmente devido ao baixo número de prescrições registradas. Importa considerar que o início precoce *versus* tardio da analgesia para o trabalho de parto não provoca diferenças clinicamente significativas nos desfechos maternos e neonatais^(42)^, e que a AF nem sempre está associada a uma experiência positiva no parto, pois poderia diminuir o sentimento de controle das mulheres. Além disso, a AF é mais dispendiosa que a ANF, pois pode atrasar o segundo estágio do trabalho de parto e aumentar a probabilidade de outras intervenções, como parto instrumental e cesariana^(33,43)^.

### Limitações do estudo

Algumas limitações deste estudo devem ser observadas. Em primeiro lugar, não foram coletadas as percepções das pacientes sobre a adequação da infraestrutura. Embora seja provável que as instalações modernas e confortáveis da maternidade impactem positivamente a experiência da paciente, isto não foi formalmente avaliado. Em segundo lugar, o estudo não explorou conhecimentos, crenças e preferências prévias da paciente sobre as diferentes modalidades terapêuticas para o alívio da dor, o que pode ter influenciado suas decisões em relação ao manejo da dor. Da mesma forma, não foram avaliadas crenças, conhecimentos, preocupações e necessidades dos profissionais de saúde em relação ao tratamento da dor. Em terceiro lugar, o estudo não avaliou a prevalência de ansiedade, depressão ou outros problemas de saúde mental que podem ter afetado a experiência da dor de parto. Em quarto lugar, o viés de memória pode ter influenciado a acurácia dos dados coletados, e, para minimizá-lo, as entrevistas foram realizadas em até 24 horas após o parto. Em quinto lugar, não foi avaliado se os plantões estavam superlotados de pacientes ou se as equipes, compostas por médicos e enfermeiros, estavam completas em quantitativo adequado para a demanda. Finalmente, os eventos adversos e a segurança relacionados à AF não foram registrados sistematicamente no questionário.

### Contribuições para a área da saúde

De acordo com a OMS, a analgesia opioide é recomendada para alívio da dor em mulheres durante o trabalho de parto e parto, juntamente com técnicas não farmacológicas. A assistência intraparto deve sempre respeitar as preferências das mulheres, considerar as evidências clínicas, a efetividade, complexidade, diversidade, equidade, aceitabilidade e viabilidade das opções de tratamento. Respeitar as escolhas da mulher culmina em priorizar abordagens individualizadas que enfatizem as relações interpessoais baseadas no diálogo entre a parturiente e profissional de saúde.

Identificamos uma elevada PP de múltiplos métodos de ANF e a sua efetividade na melhora na diminuição da intensidade da dor, assim como a efetividade da massagem, isoladamente, para reduzir a dor intensa. Deste modo, na elevada prevalência de métodos de ANF, destaca-se a atuação das enfermeiras obstétricas no cuidado à mulher durante o trabalho de parto e parto.

Comparados com as práticas analgésicas realizadas em países desenvolvidos, a PP de AF e o tempo de administração de todo tipo de analgesia aqui estudados estiveram aquém dos padrões ideais. O nível de escolaridade baixo se associou negativamente ao pedido de analgesia. Neste sentido, é mandatório compreender que a experiência dolorosa é construída a partir de fenômenos fisiológicos, culturais, emocionais e sociais que a fazem única e complexa no seu entendimento, na sua abordagem e no seu tratamento. Por isto, é necessário aprofundar o conhecimento nos fatores associados às deficiências relacionadas ao tratamento analgésico e iniciar abordagens de contenção direcionadas às pacientes, aos profissionais e ao sistema público de saúde. Programas educacionais para as pacientes, aplicados nas Unidades Básicas de Saúde durante o acompanhamento pré-natal, permitiriam conhecer as possibilidades analgésicas, facilitando sua participação ativa nas escolhas e na tomada de decisão em conjunto com as equipes de saúde. O esclarecimento e a participação ativa das pacientes poderiam aumentar a adesão dos profissionais à prescrição e diminuir o tempo para a administração da analgesia. Programas educacionais direcionados às equipes multiprofissionais são essenciais para o entendimento sobre a complexidade do manejo da dor, considerando seus diferentes componentes (físico, social, emocional e cultural), favorecendo uma abordagem individualizada que priorize o desejo da gestante e enfatizando sempre o diálogo e a colaboração entre as equipes de saúde. A proatividade das equipes multiprofissionais na avaliação e no tratamento da dor proporcionaria melhoras na avaliação clínica, na qualidade do atendimento e, provavelmente, na experiência dolorosa das parturientes. As rotinas hospitalares devem ser reavaliadas com o objetivo de desenvolver protocolos institucionais em função das gestantes, definindo critérios para pronta prescrição e implementação da analgesia de acordo com as preferências das pacientes. Finalmente, também se faz necessária uma abordagem administrativa que fortaleça a estrutura do SUS e os recursos econômicos, haja vista que um aumento na disponibilidade de analgesia provocaria uma elevação nos custos e a necessidade de contratação e treinamento eficaz de equipes multiprofissionais qualitativamente e quantitativamente adequadas.

## CONCLUSÕES

Este estudo destaca a natureza complexa do tratamento da dor durante o trabalho de parto e parto, e a subutilização das técnicas farmacológicas recomendadas para o tratamento da dor. Embora tenhamos observado uma redução significativa na proporção de pacientes com dor intensa após o tratamento com ANF e a efetividade da massagem como técnica isolada para o alívio da dor, houve um atraso na prescrição e na implementação de todo tipo de analgesia. Além disso, foi identificada, por um lado, uma associação negativa entre baixa escolaridade e o pedido de analgesia pela paciente, mas, por outro lado, que o corpo clínico nem sempre prescreveu AF a pedido da paciente, conforme recomendado pela OMS, contribuindo para a reduzida PP de AF, quando comparada com países desenvolvidos.

É crucial capacitar o corpo clínico e as pacientes sobre a prescrição apropriada de analgesia (tempo, tipo e orientação à paciente) e sobre sua diversidade e características, respectivamente. O diálogo e o trabalho colaborativo entre as equipes multiprofissionais e as pacientes são fundamentais. Ainda, há carência de disponibilidade de instrumentos multidimensionais validados para medir o impacto da dor durante o trabalho de parto e parto. Para aprofundar o conhecimento no tratamento da dor em gestantes em trabalho de parto, são necessários estudos quantitativos e qualitativos para avaliar as barreiras na avaliação e tratamento da dor, assim como desenvolver múltiplas intervenções centradas na mulher com o objetivo de melhorar a experiência dolorosa.
